# Supplementary material for: Synergistic Effects of UVB and Ionizing Radiation on Human Non-Malignant Cells: Implications for Ozone Depletion and Secondary Cosmic Radiation Exposure
Source: Biomolecules. 2025 Apr 6;15(4):536. doi: 10.3390/biom15040536 (PMC12024869; doi:10.3390/biom15040536)
Supplement: Supplementary file 1 [file biomolecules-15-00536-s001.zip › biomolecules-3499358-supplementary.pdf]

biomolecules

MDPI

Supplementary Data

Synergistic Effects of UVB and Ionizing Radiation on Human Non-Malignant Cells: Implications for Ozone Depletion and Secondary Cosmic Radiation Exposure

Angeliki Gkikoudi [1,2], Gina Manda [3], Christina Beinke [4], Ulrich Giesen [5], Amer Al-Qaaod [5], Elena-Mihaela Dragnea [3], Maria Dobre [3], Ionela Victoria Neagoe [3], Traimate Sangsuwan [6,7], Siamak Haghdoost [6,7], Spyridon N. Vasilopoulos [1], Sotiria Triantopoulou [2], Anna Georgakopoulou [1], Ioanna Tremi [1,8], Paraskevi N. Koutsoudaki [8], Sophia Havaki [8], Vassilis G. Gorgoulis [8,9,10,11,12], Michael Kokkoris [13], Faton Krasniqi [5], Georgia I. Terzoudi [2] and Alexandros G. Georgakilas [1,*]

1 DNA Damage Laboratory, Physics Department, School of Applied Mathematical and Physical Sciences, National Technical University of Athens (NTUA), Zografou Campus, 15780 Athens, Greece; angelikigkikoudi@mail.ntua.gr (A.G.); svasilopoulos@mail.ntua.gr (S.N.V.); ge19141@mail.ntua.gr (A.G.); ioannatre@med.uoa.gr (I.T.)
2 Laboratory of Health Physics, Radiobiology & Cytogenetics, Institute of Nuclear & Radiological Sciences & Technology, Energy & Safety, National Centre for Scientific Research "Demokritos", 15341 Agia Paraskevi, Greece; iro@rrp.demokritos.gr (S.T.); gterzoudi@rrp.demokritos.gr (G.I.T.)
3 Radiobiology Laboratory, "Victor Babes," National Institute of Pathology, 99-101 Splaiul Independentei, 050096 Bucharest, Romania; gina.manda@ivb.ro (G.M.); elenamihaeladragnea@gmail.com (E.-M.D.); maria.dobre@ivb.ro (M.D.); ionela.neagoe@ivb.ro (I.V.N.)
4 Bundeswehr Institute of Radiobiology, University of Ulm, Neuherbergstraβe 11, 80937 Munich, Germany; christinabeinke@bundeswehr.org
5 Physikalisch-Technische Bundesanstalt (PTB), Bundesallee 100, 38116 Braunschweig, Germany; amer.al-qaaod.ext@ptb.de (A.A.-Q.); faton.krasniqi@ptb.de (F.K.)
6 ABTE/ToxEMAC Laboratory, University of Caen Normandy, F-14050 Caen, France; traimate.sangsuwan@su.se (T.S.); siamak.haghdoost@unicaen.fr (S.H.)
7 Department of Molecular Biosciences, The Wenner-Gren Institute, Stockholm University, SE-10691 Stockholm, Sweden
8 Molecular Carcinogenesis Group, Department of Histology and Embryology, Medical School, National and Kapodistrian University of Athens, 11527 Athens, Greece; pkoutsoudaki@med.uoa.gr (P.N.K.); shavaki@med.uoa.gr (S.H.); vgorg@med.uoa.gr (V.G.G.)
9 Biomedical Research Foundation, Academy of Athens, 11527 Athens, Greece
10 Ninewells Hospital and Medical School, University of Dundee, Dundee DD2 1SG, UK
11 Faculty Institute for Cancer Sciences, Manchester Academic Health Sciences Centre, University of Manchester, Manchester M20 4GJ, UK
12 Faculty of Health and Medical Sciences, University of Surrey, Guildford GU2 7YH, UK
13 Group of Nuclear Physics, Physics Department, School of Applied Mathematical and Physical Sciences, National Technical University of Athens (NTUA), 15780 Zografou, Greece; kokkoris@central.ntua.gr
* Correspondence: alexg@mail.ntua.gr; Tel.: +30-210-7724453

S1. Irradiation setups

Cosmic radiation is a complex mixture of high-energy particles, including protons and muons, which interact with the Earth's atmosphere and contribute to the radiation environment on the ground. The stopping power of these particles in biological or tissue-equivalent materials is a crucial factor in assessing their potential biological impact. As shown in Figure 1 of the main text, protons exhibit higher stopping power at lower energies, while muons, with their broader energy spectrum, contribute significantly at higher energies. In this study, a partial simulation of the cosmic radiation field was conducted using 10 MeV protons, which have a total stopping power of approximately 5 keV/μm in tissue-equivalent material—comparable to low-energy muons. Figure 1 is

based on data from databases from NIST (https://physics.nist.gov/cgi-bin/Star/ap_table.p), LNBL to simulate muon stopping power in A-150 TEP) [1] and simulations with EXPAS to simulate both proton and muon fluxes on the ground) [2]. Different types of cells were exposed to protons or gamma rays, and UVB radiation as single stressors, and to their combination. Irradiation setups are shown in Figure S1.

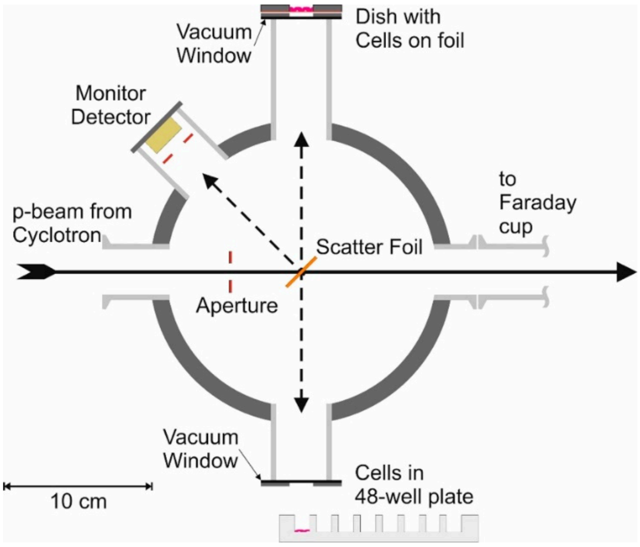

(**a**)

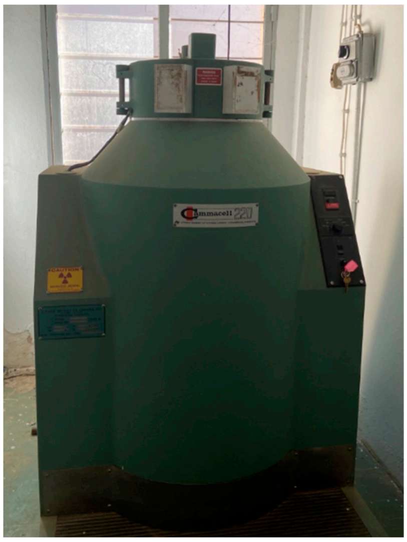

(**b**)

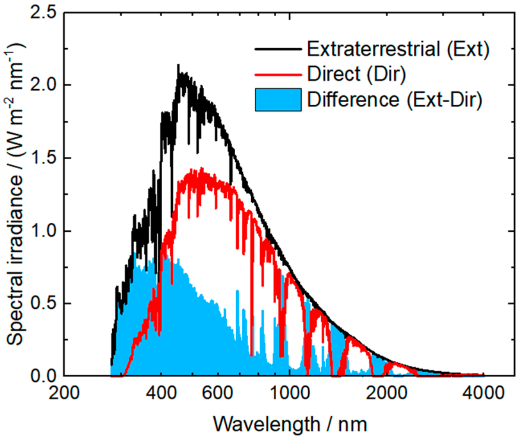

(**c**)

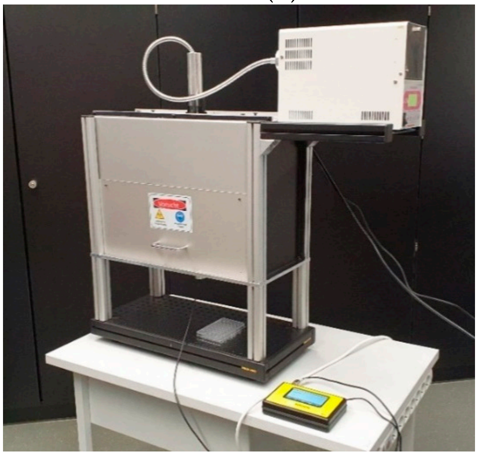

(**d**)

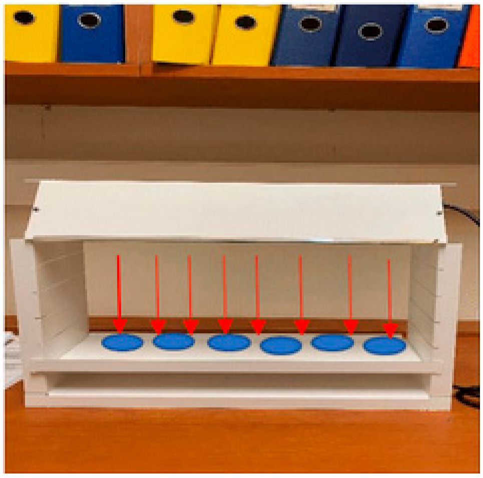

(e)

Figure S1. Irradiation setups. (a) Schematic overview of the proton irradiation setup at PTB; (b) Co^{60} source in NCSR "Demokritos; (c) The reference AM 1.5 spectra defined by the American Society for Testing and Materials (ASTM); (d) UVB spot source unit with irradiation chamber and monitor radiometer; (e) the UV Bench Lamp Model XX-15M.

S2. Stress genes in the RT² Profiler™ PCR Array Human Stress & Toxicity PathwayFinder (PAHS-003Z, Qiagen)

The expression of 84 genes involved in stress and toxicity was evaluated using the RT² Profiler™ PCR Array Human Stress & Toxicity PathwayFinder (PAHS-003Z, Qiagen, Hilden, Germany) (Table S2), and the SYBR Green chemistry on ABI7500 Fast PCR System (Thermo Fisher Scientific, Waltham, MA, USA).

Table S2. Stress genes in the RT² Profiler™ PCR Array Human Stress & Toxicity PathwayFinder (PAHS-003Z, Qiagen).

Genes		
Cell death	Apoptosis	*CASP1 (ICE), FAS, MCL1, TNFRSF10A (TRAIL-R), TNFRSF10B (DR5), TNFRSF1A (TNFR1)*
	Necrosis	*FAS, GRB2, PARP1 (ADPRT1), PVR, RIPK1, TNFRSF10A (TRAIL-R), TNFRSF1A (TNFR1), TXNL4B*
	Autophagy	*ATG12, ATG5, ATG7, BECN1, FAS, ULK1*
DNA Damage & Repair	Cell Cycle Arrest & Checkpoints	*CDKN1A (p21CIP1, WAF1), CHEK1, CHEK2 (RAD53), DDIT3 (GADD153, CHOP), HUS1, MRE11, NBN, RAD17, RAD9A*
	Other DNA Damage Responses	*ATM, ATR, DDB2, GADD45A, GADD45G, RAD51, TP53 (p53), XPC*
Unfolded Protein Response		*ATF4, ATF6, ATF6B, BBC3 (PUMA), BID, CALR, DDIT3 (GADD153, CHOP), DNAJC3, HSP90AA1, HSP90B1, HSPA4 (HSP70), HSPA5 (GRP78)*
Oxidative Stress		*FTH1, GCLC, GCLM, GSR, GSTP1, HMOX1, NQO1, PRDX1, SQSTM1, TXN, TXNRD1*

Hypoxia Signaling	*ADM, ARNT, BNIP3L, CA9, EPO, HMOX1, LDHA, MMP9, SERPINE1 (PAI-1), SLC2A1, VEGFA*
Inflammatory Response	*CCL2 (MCP-1), CD40LG, CRP, CXCL8 (IL8), IFNG, IL1A, IL1B, IL6, TLR4, TNF*
Osmotic stress	*AKR1B1, AQP1, AQP2, AQP4, CFTR, EDN1, HSPA4L (OSP94), NFAT5, SLC5A3*

S3. Additional graphs on the viability of human normal Hs27 skin fibroblasts exposed to UVB or/and protons

The impact of UVB and broad proton beams as single or combined radiations on human normal skin cells (Hs27 fibroblasts and HaCaT keratinocytes) and normal CRL 9855 was investigated using the MTS reduction assay that provides information on the number of metabolically active cells in culture, in conjunction with the LDH release assay that indicates plasma membrane alterations related to cell death by necrosis, necroptosis and late apoptosis. In the case of Hs27 cells, the impact of various UVB fluences on the viability of human normal Hs27 skin fibroblasts was investigated at 6 h and 24 h post-exposure in terms of MTS reduction and LDH release. The MTS reduction by Hs27 fibroblasts was not affected at 6 h after UVB exposure but decreased following a dose-effect relationship at fluences > 50 J/m² at 24 h (Figure S3.1-a). This effect on MTS reduction was not accompanied by an increase of LDH release (Figure S3.1-b), indicating that the decrease of the number of metabolically active Hs27 fibroblasts was not due to cell death accompanied by alterations of the plasma membrane integrity. In fact, the slight decrease of LDH release, that paralleled the decrease of MTS reduction at fluences over 100 J/m², indicated the decrease of the spontaneous (basal) LDH release due to a lower number of metabolically active cells in culture.

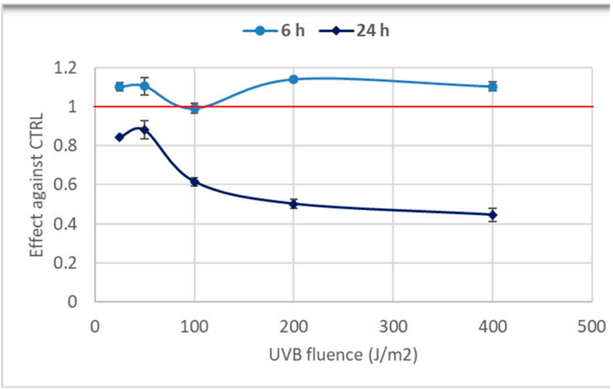

(**a**)

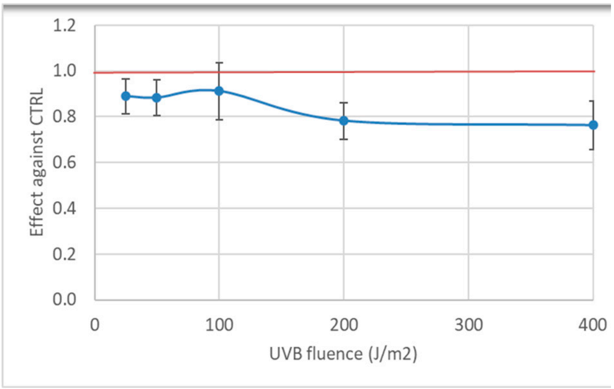

(**b**)

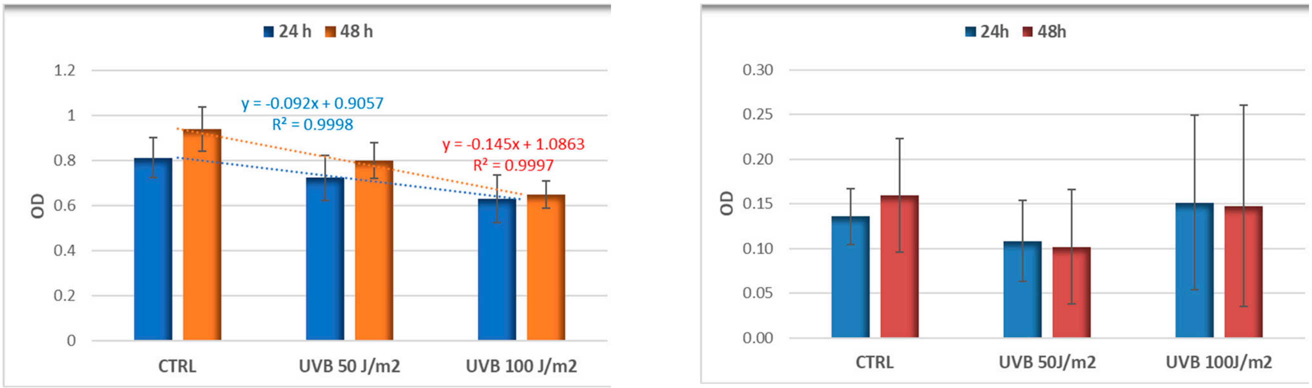

(c) **(d)**

Figure S3.1. Viability of human normal Hs27 skin fibroblasts exposed to UVB. The OD of exposed samples was divided by the mean OD value of unexposed control cells to determine the impact of UVB radiation. (a) MTS reduction by cells exposed to different UVB fluences, measured in triplicate samples at 6 and 24 hours after exposure (mean effect of UVB exposure ± SEM). The unit effect (no effect) is indicated by the red line; (b) The release of LDH by cells exposed to different UVB fluences, measured in triplicate samples 24 hours after exposure (mean effect of UVB exposure ± SEM). The unit effect (no effect) is indicated by the red line; (c) At 24 and 48 hours after exposure, the MTS decrease by cells exposed to UVB fluences of 50 J/m2 and 100 J/m2 was measured (mean OD ± SD for 7 separate experiments); (d) LDH release by cells exposed to UVB fluences of 50 J/m2 and 100 J/m2 is measured at 24 and 48 hours post-exposure (mean OD ± SD for 7 independent experiments), along with the trendlines at 24 and 48 hours (blue and red lines), together with their equations.

We further investigated in more detail, in 7 independent experiments, the effect of UVB at fluences of 50 J/m^2 and 100 J/m^2 on the viability of Hs27 fibroblasts at 24 h and 48 h post-exposure. MTS reduction decreased following a dose-effect relationship (Figure S3.1-c). While 100 J/m^2 UVB decreased MTS reduction both at 24 h and 48 h after exposure ($p<0.05$), 50 J/m^2 UVB inflicted damages slowly, the changes vs CTRL being significant only at 48 h ($p<0.05$). The effects of 50 J/m^2 and 100 J/m^2 were statistically different both at 24 h ($p<0.01$) and 48 h ($p<0.001$). Of note is that cells exposed to 100 J/m^2 did not proliferate anymore between 24 h and 48 h post-exposure to UVB, as no difference in MTS reduction were registered. The decreased MTS reduction was not accompanied by significant changes of LDH release (Figure S3-d), indicating that cells exposed to the investigated UVB fluences did not die by massive necrosis/necroptosis or late apoptosis that involve alteration of the plasma membrane integrity.

When Hs27 fibroblasts were exposed to protons of various doses (0.25 Gy to 1 Gy), no significant changes of MTS reduction at 24 h after irradiation were detected, neither when cells were placed in the bottom plate or in the upper dish (Figure S3.2-a). Exposure of cells to 0.5 Gy protons did not significantly alter the viability of fibroblasts in terms of MTS reduction (Figure S3.2-b) or LDH release (Figure S3.2-c). Altogether, experimental data show that protons did not alter the viability of Hs27 fibroblasts in the investigated dose range, no matter if cells were exposed to protons in the bottom plate or in the upper dish.

A more detailed analysis was performed on Hs27 fibroblasts exposed to 0.5 Gy protons in the bottom plate. Two controls were used for comparison: a control kept in the biology laboratory (CTRL), also used as reference for UVB exposure, and a control in the irradiation facility (IrrCTRL). Control cells kept in the irradiation room (IrrCTRL), had a small decrease of MTS reduction compared to the laboratory CTRL (Figure S3.2-b), indicating that they might have been exposed and slightly harmed by the background radiation (14 mGy per Gy of proton dose delivered to cells). The exposure of cells to 0.5 Gy protons (Figure S3.2-b) decreased the MTS reduction as compared to CTRL,

especially at 48 h post-exposure ($p<0.05$), but not at 24 h when cells appeared to be mostly affected by the background radiation. Altogether, experimental data indicated that 0.5 Gy protons inflicted a slight reduction of the number of metabolically active Hs27 fibroblasts at 48 h after their exposure, and that cells seemed to be slightly affected also by the background radiation in the irradiation facility.

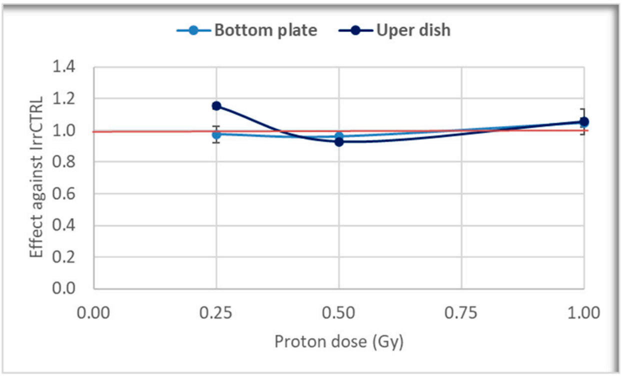

(**a**)

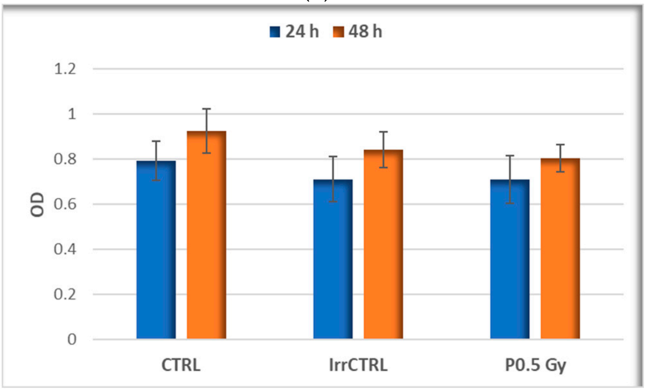

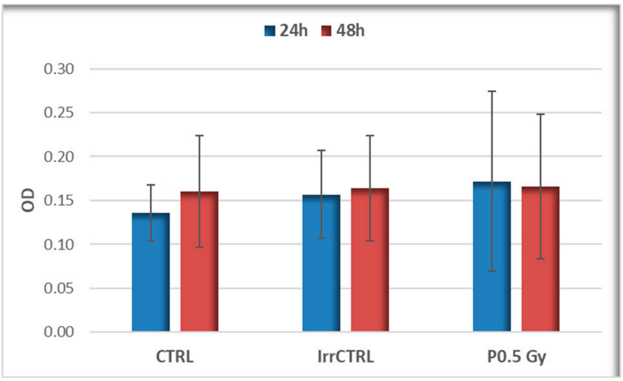

(**b**) (**c**)

Figure S3.2. MTS reduction by human normal Hs27 skin fibroblasts exposed protons. The effect of proton exposure was calculated as OD of exposed samples divided by the mean OD value of unexposed control cells kept in the irradiation facility (IrrCTRL). **a.** Cells exposed to various doses of protons, and analysed against the IrrCTRL at 24 h post-exposure on triplicate samples (mean effect of proton exposure ± SEM). The red line designates the unit effect (no effect); **b.** MTS reduction at 24 h and 48 h after exposure of cells to 0.5 Gy protons (mean OD ± SD for 7 independent experiments); **c.** LDH release at 24 h and 48 h after exposure of cells to 0.5 Gy protons (mean OD ± SD for 7 independent experiments).

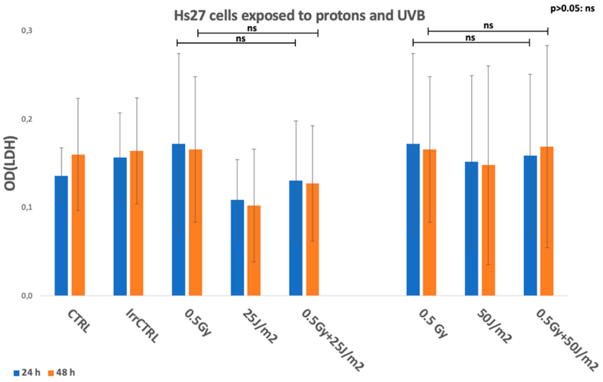

Figure S3.3. LDH release on Hs27 fibroblasts at 24 h and 48 h after exposure to 0.5 Gy protons, and thereafter, within approx. 20 min, to UVB (50 J/m² or 100 J/m²) (mean effect ± SD for 3 independent experiments).

S4. Additional graphs on the viability of human normal HaCaT skin keratinocytes exposed to UVB and/or protons

UVB exposure of HaCaT cells had major effects on MTS reduction at 6 h post-exposure, but drastically decreased MTS reduction at 24 h after irradiation at fluences ≥ 50 J/m2 (Figure S4-a), with a significant increase of LDH release at fluences ≥ 100 J/m2 (Figure S4-b). Results indicate that UVB decreased slowly the number of metabolically active cells within 24 h post-exposure, and this was partly due to cell death with alterations of the plasma membrane.

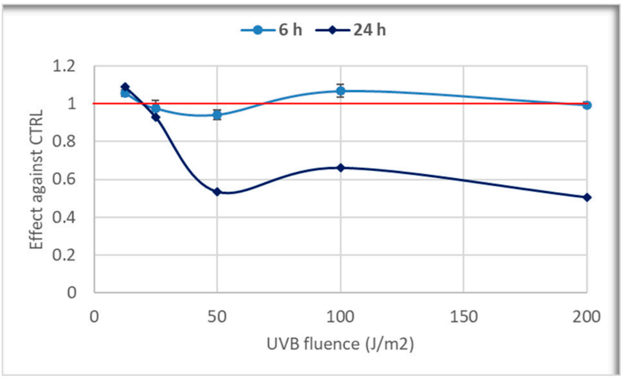

(a)

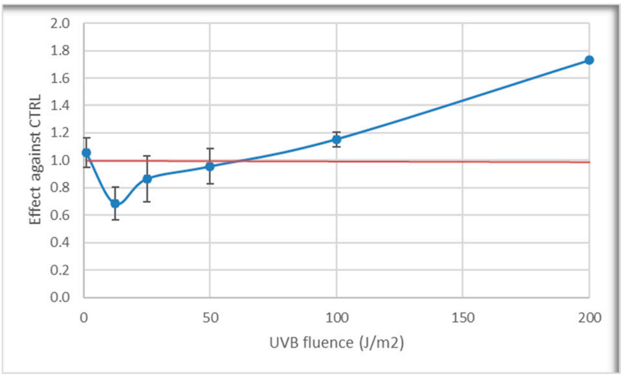

(b)

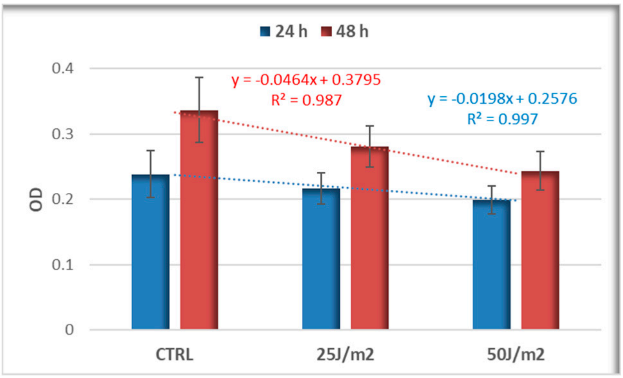

(c)

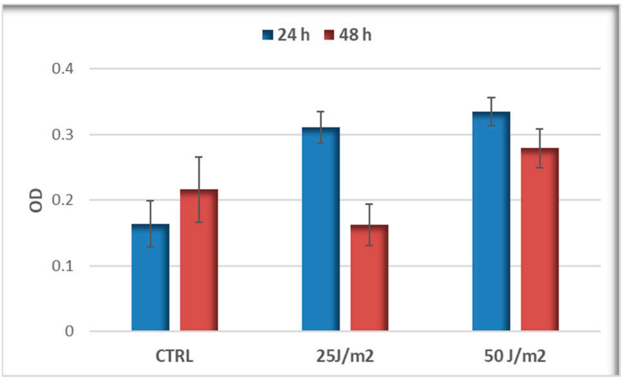

(d)

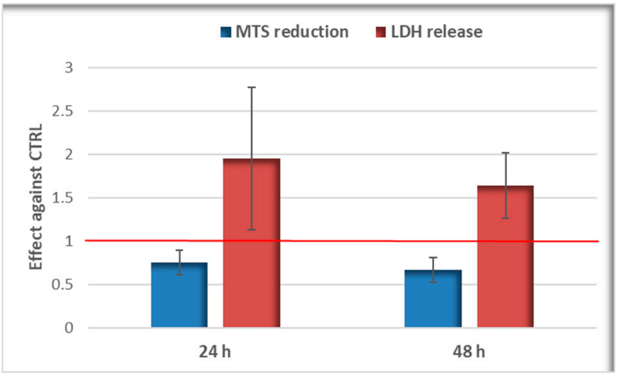

(e)

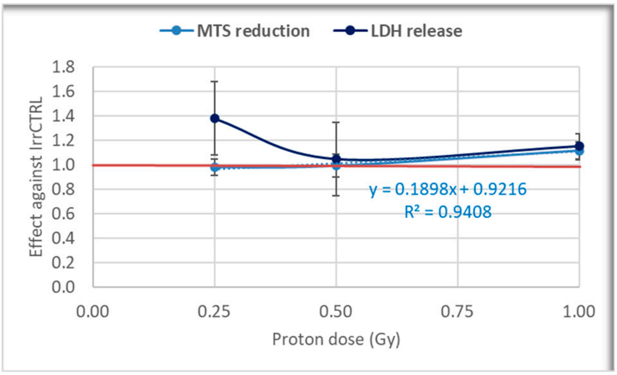

(f)

Figure S4.1. Viability of human normal HaCaT keratinocytes exposed to UVB. The effect of UVB exposure was calculated as OD of exposed samples divided by the mean OD value of unexposed control cells (CTRL). a. MTS reduction by cells exposed to various UVB fluences, assessed at 6 h and 24 h post-exposure in triplicate samples (mean effect of UVB exposure ± SEM). The red line designates the unit effect (no effect). Trendlines at 24 h (blue line) and 48 h (red line), and their equations are presented; e. MTS reduction by cells exposed to 50 J/m² UVB, assessed at 24 h and 48 h post-exposure (mean OD ± SD for 7 independent experiments); b. LDH release by cells exposed to various UVB fluences, assessed at 24 h post-exposure in triplicate samples (mean effect of UVB exposure ± SEM). The red line designates the unit effect (no effect); c. MTS reduction by cells exposed to UVB fluences of 25 J/m² and 50 J/m², assessed at 24 h and 48 h (mean OD ± SD for 3 independent experiments). Trendlines at 24 h (blue line) and 48 h (red line), and their equations are presented; d. LDH release by cells exposed to UVB fluences of 25 J/m² and 50 J/m², assessed at 24 h and 48 h (mean OD ± SD for 3 independent experiments). f. MTS reduction by human normal HaCaT keratinocytes exposed to various doses of protons (mean effect ± SD for 3 independent experiments). The effect of UVB exposure was calculated as OD of exposed samples divided by the mean OD value of unexposed control cells kept in the biology laboratory, outside the irradiation facility (CTRL). The effect of proton exposure was calculated as OD of exposed samples divided by the mean OD value of unexposed control cells kept in the irradiation facility (IrrCTRL). The red line designates the unit effect (no effect).

A more detailed analysis of 3 independent experiments on the effect of UVB 25 J/m² and 50 J/m2 (Figure S4-c) showed a dose-effect relationship of MTS reduction by HaCaT keratinocytes both at 24 h and 48 h after exposure ($p<0.05$). The decrease of MTS reduction was accompanied by a fluence-independent increase of LDH release at 24 h, that persisted also at 48 h only in the case of cells exposed to 50 J/m² UVB (Figure S4-d), indicating that this higher fluence was persistently cytotoxic, as shown also in Figure S4-e with data from 7 independent experiments.

When HaCaT keratinocytes were exposed to protons in the dose range of (0.25 – 1) Gy no significant changes of MTS reduction were registered (Figure S4-e).

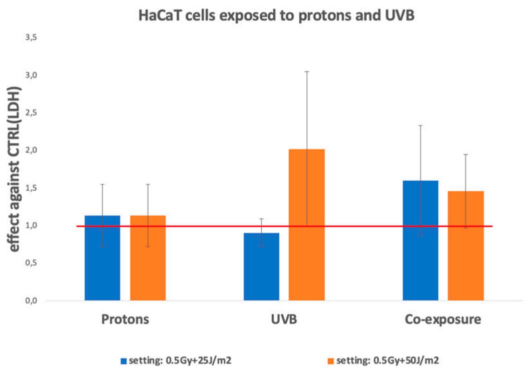

Figure S4.2. LDH release on HaCaT keratinocytes at 48 h post-exposure (mean effect ± SD for 3 independent experiments);

S5. Additional graphs on the viability of human CRL9855 monocytes exposed to UVB and/or protons

Exposure of human CRL 9855 monocytes to UVB drastically decreased the MTS reduction at 24 h after the exposure of cells, 50 J/m² being the first investigated fluence at which a significant effect (effect < 0.8) was registered (Figure S5-a).

While only a slight decrease in MTS reduction was triggered by 0.25 Gy protons, 0.5 Gy protons induced a stronger effect in term of decrease of metabolically active cells ($p<0.05$) Figure S5-b).

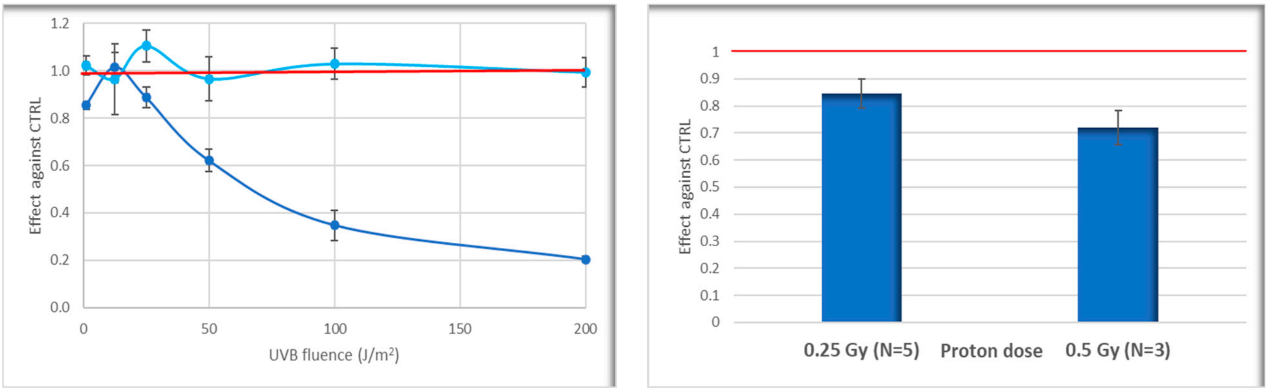

(**a**) (**b**)

Figure S5. The effect of either UVB (a) or proton (b) irradiation on the MTS reduction by normal CRL 9855 monocytes. The effect of proton exposure was calculated as OD of exposed samples divided by the mean OD value of unexposed control cells kept in the biology laboratory, outside the irradiation facility (CTRL). Results are presented as mean exposure effect ± SEM (a. triplicate samples) or as mean exposure effect ± SD (b. 3-5 independent experiments).

S6. Additional data regarding γH2AX Immunofluorescence assay

Graphs of DNA damage and repair HS27 and HaCaT cells exposed to UVB first and within 20 min to protons (the vice-versa set up)

To examine whether there is a statistically significant difference in results obtained from the two irradiation set-ups we exposed both cell lines in the same doses and UVB fluences but starting with UVB exposure and the performed the γH2AX IF assay, 4 and 24 hours after the treatment. Hs27 fibroblasts subjected to 0.5 Gy protons administered as single radiation showed a small increase in the number of γH2AX foci per nucleus after 4 hours, but a considerable decrease at the 24 hour time point (Figure S6-a). The γH2AX foci increased after 4 hours and then decreased at 24 hours in cells exposed to 100 J/m² UVB (Figure S6-a), while when 100 J/m² and 0.5 Gy protons were delivered together, there were significantly more γH2AX foci per nucleus than when either treatment was used alone. The notable rise in γH2AX foci indicates a synergistic interaction between protons and UVB that intensifies DNA damage. Hs27 fibroblasts exposed to 0.5 Gy protons alone showed a very low percentage of pan-nuclear-stained cells with γH2AX staining at 4 and 24 hours after irradiation (Figure S6-b), indicating that there was little to no global DNA damage throughout the nucleus. In contrast, 100 J/m² UVB caused a significantly greater percentage of pan-nuclear γH2AX staining (about 50% of cells stained at 4 hours), which slightly decreased by 24 hours (Figure S6-b). This suggests that UVB alone causes significant pan-nuclear DNA damage, which gradually diminishes as a result of active repair processes. The highest percentage of pan-nuclear staining was observed in the samples co-exposed to 100 J/m² and 0.5 Gy at the 24 h time point.

There were no statistically significant differences between the two co-exposure scenarios at any of the time points that were examined (4 and 24 hours) ($p>0.05$). This indicates that, despite the observed distinct trends in DNA damage and repair, the differences in γH2AX foci between the two exposure sequences were not statistically significant, and could be attributed currently to experimental variability mostly. Thus, whether protons come before UVB or vice versa, the overall response to combined radiation exposure appears to produce similar levels of DNA damage and repair kinetics, though the latter may have a greater effect.
HaCaT cells were first exposed to UVB, and then to protons, were analyzed at 6 h and 24 h post-exposure. At 6 hours after exposure, there was a discernible rise in γH2AX foci,

however, after 24 hours, there were fewer foci counted (Figure S6-c). The γH2AX foci increased moderately when cells were exposed to 50 J/m² of UVB alone with a of peak foci at 6 hours and a decrease at 24 hours (Figure S6-c). Crucially, there was a synergistic effect from the combined exposure to 50 J/m² UVB and 0.5 Gy protons with the greatest number of γH2AX foci was seen at 6 hours (Figure S6-c). At the 6-hour time point, the elevated γH2AX foci showed that this exposure setting caused significantly more DNA damage than either treatment. While the number of foci decreased after 24 hours (Figure S6-c), it was still greater than the amounts seen in individual exposures but did not differ significantly from cells exposed to protons alone ($p>0.05$).
Proton irradiation may have caused limited pan-nuclear DNA damage, as evidenced by the relatively low percentage of pan-nuclear stained cells that increased slightly at 24 hours (Figure S6-d). At 6 hours, when the damage response was reportedly at its highest, the percentage of pan-nuclear stained cells increased more noticeably in response to exposure to 50 J/m² UVB with a discernible decrease in pan-nuclear staining at 24 h (Figure S6-d). The most significant outcome, however, was observed when cells were exposed to 0.5 Gy protons after 50 J/m² UVB exposure. At 6 h, this combined exposure significantly increased the percentage of pan-nuclear stained cells, surpassing the levels brought on by either treatment alone even after 24 hours (Figure S6-d).
The results of comparing the two co-exposure scenarios revealed statistically significant differences at the two time points (4 and 24 hours) ($p<0.05$). When DNA damage was strongly and immediately increased when cells were first exposed to protons and then UVB. Over time, particularly when the higher UVB dose (50 J/m2) was applied, this damage was sustained. According to this, proton irradiation sensitizes HaCaT keratinocytes, increasing their susceptibility to further UVB-induced harm. The initial response to UVB-induced DNA damage was less obvious when proton exposure was followed by UVB exposure, however, the subsequent proton exposure, which led to a significant increase in γH2AX foci, amplified the damage. These findings suggest that although UVB radiation by itself can cause DNA damage, the presence of protons intensifies the effect and causes more long-lasting harm.
<u>γH2AX immunofluorescence assay in VH10 cells</u>

As mentioned in the main text, γH2AX immunofluorescence assay was performed in a different fibroblast cell line (VH10 cells). Fluorescence images are presented in Figure S6e.

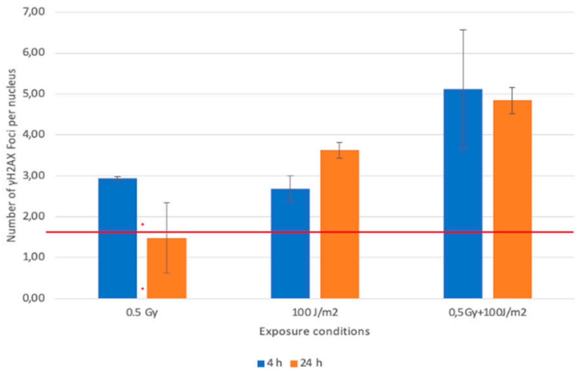

(a)

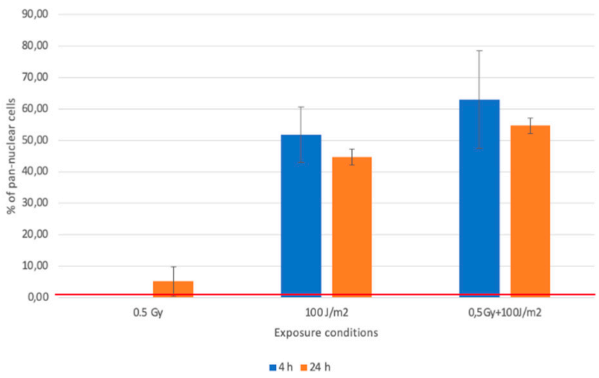

(b)

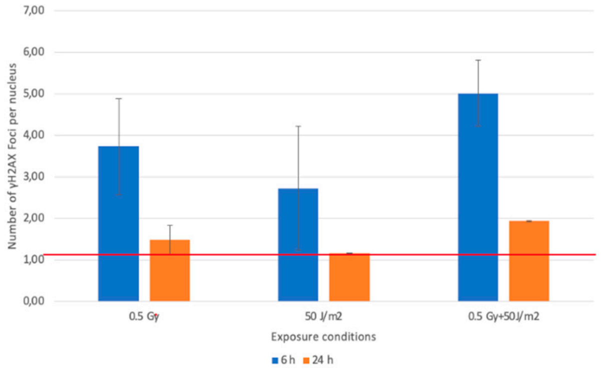

(c)

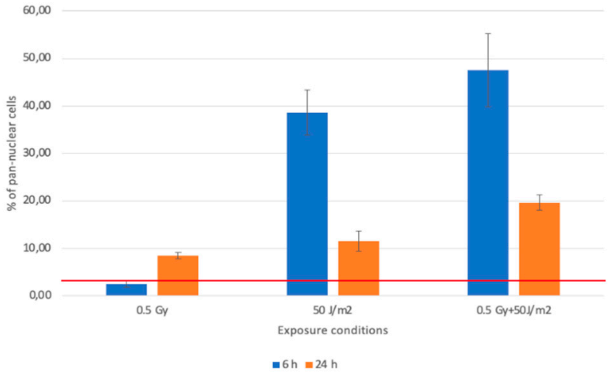

(d)

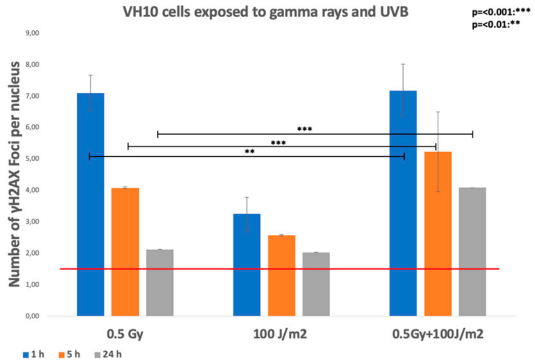

(e)

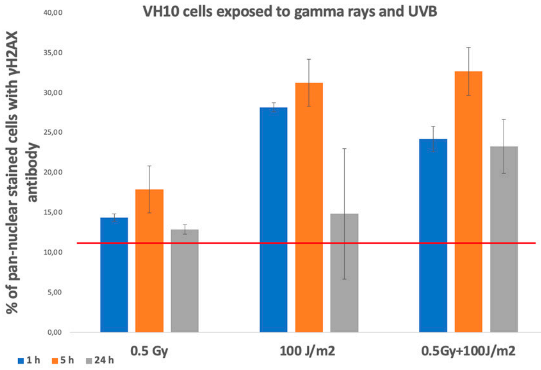

(f)

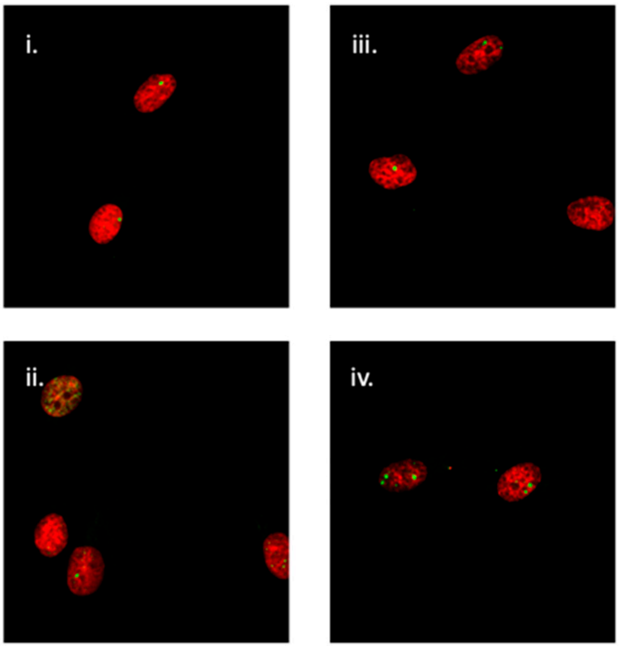

(g)

Figure S6. γH2AX staining in human non-malignant cells. Results are presented as mean effect (value) ± SD for 2 independent experiments. The red line designates the mean value in control samples. (a) γH2AX foci per nucleus in Hs27 cells at 4 h and 24 h after exposure to 0.5 Gy protons or UVB (100 J/m^2) as single stressors, or to combined challenges (UVB first, and protons 15 min thereafter; (b) Pan nuclear γH2AX staining in Hs27 cells at 4 h and 24 h after exposure to 0.5 Gy protons or UVB (100 J/m^2) as single stressors; (c) γH2AX foci per nucleus in HaCaT cells at 6 h and

24 h after exposure to 0.5 Gy protons or UVB (50 J/m^2) as single stressors, or to combined challenges (UVB first, and protons 15 min thereafter; (d) Pan nuclear γH2AX staining in HaCaT cells at 6 h and 24 h after exposure to 0.5 Gy protons or UVB (50 J/m^2) as single stressors, or to combined challenges (UVB first, and protons 20 min thereafter); (e) γH2AX foci per nucleus in VH10 cels at 1 h, 5 h and 24 h after exposure to 0.5 Gy gamma rays or or 100 J/m^2 UVB as single stressors, or to combined challenges (protons first, and UVB 20 min thereafter) ($stdev_{control}$=0.47 foci/nucleus); (f). Pan nuclear γH2AX staining in VH10 cells at 1 h, 5 h and 24 h after exposure to 0.5 Gy gamma rays or UVB 100 J/m^2 as single stressors, or to combined challenges (gamma rays first, and UVB 20 min thereafter) ($stdev_{control}$=0.49%); (g) γH2AX staining in VH10 cells (i) unirradiated, (ii) exposed to 100 J/m^2 UVB, (iii) exposed to 0.5 Gy gamma rays and (iv) co-exposed to 0,5 Gy gamma rays and 100 J/m^2 UVB all 24 h post-exposure (red signal: cell nucleus, green signal: γH2AX foci).

S7. Dose-response relationship generated for dicentric induction after proton exposure

For the analysis of the dose-response relationship of proton-induced dicentrics after 0.0 Gy, 0.1 Gy, 0.25 Gy, 0.5 Gy, 0.75 Gy and 1.0 Gy replicate cultures have been set up per dose in two experiments (two donors; in total four cell cultures per dose point). A plate control has been set up for each dose. For each culture, dose response data collected for neutron-gamma background (plate control) were used to calculate the "only-proton"-induced dicentric frequency by subtraction (CdicP+NG/cell – CdicNG/cell = dicP-calc/cell). Dose-response data were plotted using Sigma Plot 14.0 software.

The standard u-test goodness of fit described by Papworth and adopted by Savage (1970) using Biodosetool version 3.6.1 (Hernández et al. 2023) was used to test the distribution of observed dicentrics (CdicP+NG) for Poisson probabilities. If the magnitude of the u-value is greater than +1.96 or less than -1.96, the over- or under-dispersion of dicentrics is significant at the 5 % confidence level and indicates a heterogeneous exposure.

The dose-response curve and its 95% confidence interval of proton-induced dicentrics (including the neutron-gamma background; CdicP+NG), evaluated by semi-automated scoring was fitted to a linear dose-response model (Y=c+a*D) using Biodosetool version 3.6.1[3].

Table S7 summarizes the frequencies and distributions of dicentrics in human peripheral lymphocytes induced by 0.1 Gy to 1.0 Gy protons (CdicP+NG) and by the neutron-gamma background (CdicNG) as well as the calculated yields for only proton-induced dicentrics (CdicP+NG - CdicNG = dicP-calc). With increasing doses, the exposure time increased from about 5 min (0.1 Gy protons) to about 50 min (1.0 Gy protons). The dicentric yields observed due to the proton plus background irradiation as well as induced by the neutron-gamma background is increasing with increasing proton dose from 0.1 Gy up to 1.0 Gy (Table S2, Figure S7). However, the neutron-gamma induced dicentric frequency rises slightly from 0.0008 to 0.0080 CdicNG/cell (Figure X, fit of neutron-gamma bg), whereas the frequency of the proton plus neutron-gamma background induced dicentrics increase from 0.0058 to 0.0732 CdicP+NG/cell. Accordingly, the mathematically calculated dicentric formation due to the proton irradiation also increases up to 1.0 Gy from 0.0050 to 0.0652 CdicP-calc/cell.

Table S7. Numbers and distribution of dicentrics and dicentric yields (means of two donors) in human lymphocytes irradiated with 10 MeV protons (4.7 keV/μm).

proton dose (Gy)	cells evaluated	[a]Cdic $_{P+NG}$	Cdic$_{P+NG}$/ cell	SE Cdic$_{P+NG}$ / cell	Cdic$_{P+N}$/cell					var/ mean	[b]u-value	cells evaluated for Cdic$_{NG}$ (plate co)	Cdic$_{NG}$	Cdic$_{NG}$/ cell	SE dic$_{NG}$	[c]dic$_{P\text{-}calc}$/ cell
					0	1	2	3	4							
0.0	16494	13	0.0008	0.0002	16481	13	0	0	0	1.00	-0.07	0	/	0.0000	0.00E+00	0.0008
0.1	7240	42	0.0058	0.0009	7199	40	1	0	0	1.04	**2.56**	6515	5	0.0008	4.25E-06	0.0050
0.25	8268	98	0.0119	0.0012	8171	96	1	0	0	1.01	0.56	7964	18	0.0023*	5.97E-06	0.0096
0.5	12107	458	0.0378	0.0018	11670	418	17	2	0	1.06	**4.88**	18745	48	0.0026*	2.70E-06	0.0353
0.75	3617	180	0.0498	0.0037	3452	151	13	1	0	1.13	**5.47**	4594	12	0.0026*	1.11E-05	0.0472
1.0	4374	320	0.0732	0.0041	4077	274	23	0	0	1.07	**3.32**	5151	41	0.0080*	1.73E-05	0.0652

[a]Cdic$_{P+NG}$: dic induced by protons and neutron-gamma (NG) background in irradiation hall = plate co (control cells in well plate not exposed to proton beam, but to NG background radiation)

[b]u-value > 1,96: dicentrics are overdispersed (bold), 0 Gy: lab control (cells never positioned in the irradiation hall)

[c]dic$_{P\text{-}calc}$: dicentric yield induced by only proton irradiation calculated by subtraction of dic$_{NG}$ from dic$_{P+NG}$

* significantly different from the analzing laboratory`s reference data of unexposed cells and from the PTB laboratory control

The dicentric frequencies in the plate controls, except for the plate control of 0.1 Gy protons (p>0.001), were significantly different (p<0.001) from the previously established reference value of the analyzing laboratory (0.0005 Cdic/cell), as well as from the laboratory control (Table S2, 0.0 Gy: 13 dicentrics in 16494 cells, 0.0008 Cdic/cell). The dicentric yield observed due to the proton plus background irradiation is known to increase with increasing proton dose and fits to a linear model (Figure S7a and FigureS7b).

Dose-response relationships (linear fits, SigmaPlot version 14.0) of dicentrics obtained after proton irradiation of human peripheral blood lymphocytes with the 10 MeV proton beam (LET 4.7 keV/µm). Solid line: dicentric yield after proton irradiation including neutron-gamma induced dicentrics; dashed line: neutron-gamma background (bg) induced dicentric yield; dashed/dotted line: proton induced dicentrics yield after subtraction of the neutron-gamma background. Error bars: standard errors of the means.

However, for further evaluations the proton induced dicentric frequencies were considered including the background effect in order to prevent uncertainties due to the mathematical subtraction of dicentrics induced by the neutron gamma background.
The distribution of dicentrics was analyzed only for the dicentric induction by protons including the neutron-gamma background, because neutron-gamma induced dicentrics cannot be distinguished from proton-induced aberrations. The u-values of dicentric distributions were > +1.96, except for 0.25 Gy (u = 0.56). Thus, except for the 0.25 Gy proton dose, the dicentric distribution does not follow a Poisson distribution. However, in case of 0.25 Gy proton dose, only one more cell with two dicentrics would lead to an u-value <5.0. These results indicate a heterogeneous proton exposure of cells.
Data of the dose-response to proton irradiation including the neutron-gamma background effect were fitted to a linear model using Biodosetool software version 3.6.1 [3] as follows: Yield = 0.0007 (+/- 0.0005) + 0.0675 (+/- 0.0052)*D, where Y is the yield of dicentrics (CdicNG/cell) and D is the dose. The p value for the alpha coefficient (p of alpha = 0.00041; p<0.005) is statistically significant and the coefficient of determination (r2 = 0.9875) shows a strong correlation between dicentric yield and radiation dose. The results imply that the linear model is suitable and properly fitted here.

The dicentric yield in cells exposed to 0.5 Gy protons followed immediately by 400 J/m ² UVB was also examined but the metaphase yield was too low to be reliably analyzed for dicentrics, so no fold-induction of dicentric formation has been determined.

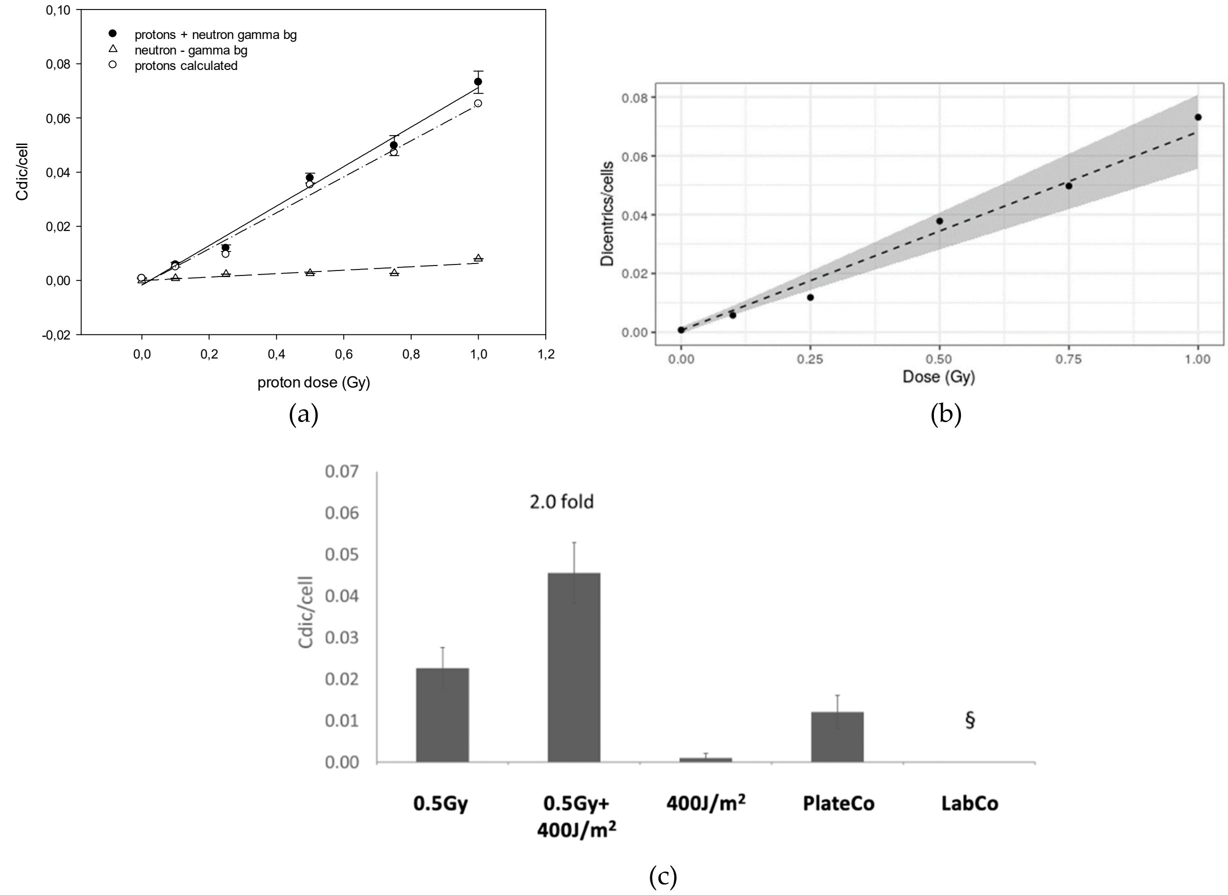

Figure S7. (a) Dose-response relationships (linear fits, SigmaPlot version 14.0) of dicentrics obtained after proton irradiation of PBMCs with the 10 MeV proton beam (LET 4.7 keV/µm). Solid line: dicentric yield after proton irradiation including neutron-gamma induced dicentrics; dashed line: neutron-gamma background (bg) induced dicentric yield; dashed/dotted line: proton induced dicentrics yield after subtraction of the neutron-gamma background. Error bars: standard errors of the means. (b) Dose-response curve and its 95% confidence interval (grey area) of 10 MeV protons (LET 4.7 keV/µm) induced dicentrics (CdicP+NG), evaluated by semi-automated scoring using DCScore (MetaSystems, Germany); linear fit using Biodosetool version 3.6.1 [3]; (c) Dicentric frequencies in PBMCs induced by 0.5 Gy protons, 400 J/m² UVB, the combined exposures (protons + UVB), neutron-gamma background (plate control) as well as observed in the PTB laboratory control. In total, four 4 cultures from 2 independent experiments have been analyzed per condition each represented by one column. §: no dicentrics observed.

S8. Combined exposures of PBMCs (additional data)

The time for 0.5 Gy proton exposure was about 25 min including transport from the laboratory to the irradiation hall and back, and 71.4 or 15.4 seconds, respectively, for UVB irradiation (400 J/m^2 or 100 J/m^2) within the laboratory. Tables S8a and b summarize the data collected after 0.5 Gy proton, 100 or 400 UVB J/m^2 UVB and combined exposures (0.5 Gy protons followed immediately by UVB exposure or vice versa) as well as in accompanying controls (CTRL and IrrcTRL). Dicentric frequencies determined in all four cell cultures are summarized in Table S8a and mean values per experiment as well as fold changes of dicentric frequencies are shown in Table S8b.

Table S8. Dicentric chromosomes in exposed PBMCs. (a) Evaluated cells, dicentric numbers (Cdic), dicentric yields (Cdic/cell) and standard deviations calculated based on four lymphocyte cultures (two cultures per experiment). Lymphocytes were exposed to protons or UVB irradiation only or to combined exposures (protons followed by UVB or vice versa). (b) Means of dicentric yields (Cdic/cell) of four cultures per irradiation condition and calculation of fold-change of dicentric yields for combined exposures compared to proton-only exposures. (c) Means of dicentric yields (Cdic/cell) of four cultures per irradiation condition and calculation of fold-change of dicentric yields for combined exposures compared to proton-only exposures.

(a)

experiments applying 100 J/m^2 UVB						experiments applying 400 J/m^2 UVB					
irradiation	** exp. #	MP	***Cdic	Cdic/cell	SD	irradiation	[b]exp. #	MP	[c]Cdic	Cdic/cell	SD
0.5 Gy protons	1A	2083	86	0.0413	0.1249	0.5 Gy protons	1A	1229	26	0.0212	0.0043
	1B	3080	142	0.0461			1B	964	24	0.0249	
	2A	271	89	0.3284			2A	878	25	0.0285	
	2B	2467	82	0.0332			2B	942	16	0.0170	
100 J/m2 UVB + 0.5 Gy protons	1A	1638	87	0.0531	0.0056	400 J/m2 UVB + 0.5 Gy protons	1A	only few MP	nd	nd	nd
	1B	1656	88	0.0531			1B				
	2A	1106	45	0.0407			2A				
	2B	1287	56	0.0435			2B				
0.5 Gy protons + 100 J/m^2 UVB	1A	2832	195	0.0689	0.0079	0.5 Gy protons + 400 J/m^2 UVB	1A	464	21	0.0453	0.0060
	1B	2289	108	0.0472			1B	1046	43	0.0411	
	2A	3120	171	0.0548			2A	488	27	0.0553	
	2B	3612	195	0.0540			2B	no MP	nd	nd	
100 J/m^2 UVB	1A	975	1	0.0010	0.0004	400 J/m^2 UVB	1A	517	0	0.0000	0.0011
	1B	1191	0	0.0000			1B	390	0	0.0000	
	2A	1202	1	0.0008			2A	439	1	0.0023	
	2B	1256	1	0.0008			2B	677	1	0.0015	
[a]plate control	1A	3680	9	0.0024	0.0005	*plate control	1A	320	2	0.0063	0.0032
	1B	3956	10	0.0025			1B	no MP	nd	nd	
	2A	3498	5	0.0014			2A	877	12	0.0137	
	2B	4145	7	0.0017			2B	1532	19	0.0124	
laboratory control (PTB)	1A	3151	3	0.0010	0.0003	laboratory control (PTB)	1A	1368	0	0.0000	0.0000
	1B	3893	5	0.0013			1B	790	0	0.0000	
	2A	4262	2	0.0005			2A	447	0	0.0000	
	2B	3963	2	0.0005			2B	778	0	0.0000	

[a]Plate control: to determine dicentric yield induced by neutron-gamma background radiation of proton beam
[b]Two cultures (A, B) per each experiment (1, 2)
[c]Cdic: dicentric chromosomes confirmed upon semi-automated dicentric evaluation with DCScore (Metasystems, Germany)
MP: metaphases, nd: not determined

(b)

experiments applying 100 J/m2 UVB					experiments applying 400 J/m2 UVB				
irradiation	MP	Cdic	Cdic/cell	fold change compared to proton IR only	irradiation	MP	Cdic	Cdic/cell	fold change compared to proton IR only
0.5 Gy protons	10342	399	0.0386*	/	0.5 Gy protons	4013	91	0.0227*	/
100 J/m2 UVB + 0.5 Gy protons	5634	276	0.0490*	1.27	400 J/m2 UVB + 0.5 Gy protons	only few MP	nd	nd	nd
0.5 Gy protons + 100 J/m2 UVB	11853	669	0.0564*	1.46	0.5 Gy protons + 400 J/m2 UVB	1998	91	0.0455*	2.00
100 J/m2 UVB	4624	3	0.0006	/	400 J/m2 UVB	2023	2	0.0010	/
[a]Plate control	15279	31	0.0020*	/	*plate control	2729	33	0.0121*	/
laboratory control (PTB)	15269	12	0.0008	/	laboratory control (PTB)	3383	0	0.000	/

[a]Plate control: to determine dicentric yield induced by neutron-gamma background radiation of proton beam
*Radiation induced dicentric frequency significant different from laboratory control (PTB) as well as from the reference value of the analyzing laboratory (<0.001)
MP: metaphases, nd: not determined

(c)

experiments applying 100 J/m2 UVB					experiments applying 400 J/m2 UVB				
irradiation	MP	Cdic	Cdic/cell	fold change compared to proton IR only	irradiation	MP	Cdic	Cdic/cell	fold change compared to proton IR only
0.5 Gy protons	10342	399	0.0386*	/	0.5 Gy protons	4013	91	0.0227*	/
100 J/m2 UVB + 0.5 Gy protons	5634	276	0.0490*	1.27	400 J/m2 UVB + 0.5 Gy protons	only few MP	nd	nd	nd
0.5 Gy protons + 100 J/m2 UVB	11853	669	0.0564*	1.46	0.5 Gy protons + 400 J/m2 UVB	1998	91	0.0455*	2.00
100 J/m2 UVB	4624	3	0.0006	/	400 J/m2 UVB	2023	2	0.0010	/
[a]Plate control	15279	31	0.0020*	/	*plate control	2729	33	0.0121*	/
laboratory control (PTB)	15269	12	0.0008	/	laboratory control (PTB)	3383	0	0.000	/

[a]Plate control: to determine dicentric yield induced by neutron-gamma background radiation of proton beam
*Radiation induced dicentric frequency significant different from laboratory control (PTB) as well as from the reference value of the analyzing laboratory (<0.001)
MP: metaphases, nd: not determined

In Figure S8 and Table S8c dicentric frequencies induced by 0.5 Gy protons, 100 J/m² UVB (a) or 400 J/m2 UVB (b), combined exposures (protons + UVB and vice versa), IrrCTRL as well as observed in CTRL samples are shown for four 4 cultures from 2 independent experiments.

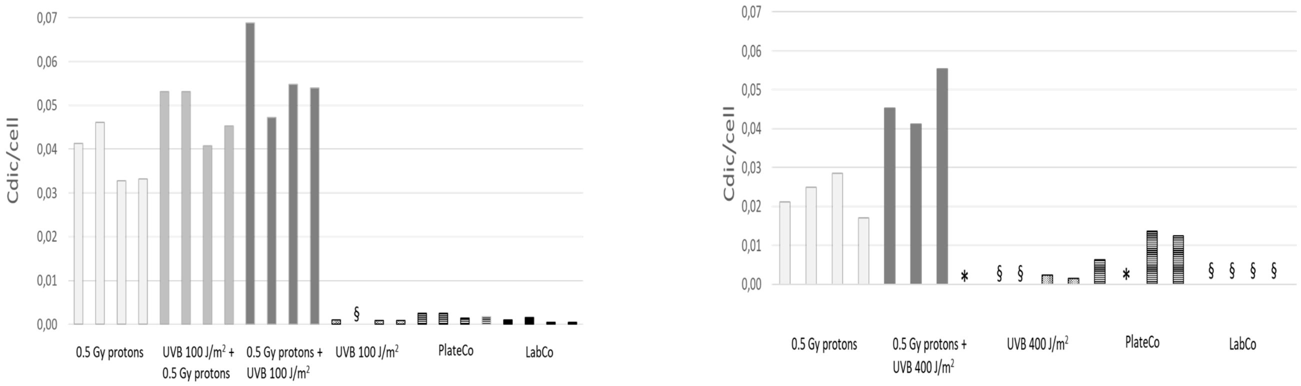

(**a**) (**b**)

Figure S8. Dicentric frequencies induced by 0.5 Gy protons, 100 J/m² UVB (a) or 400 J/m² UVB (b), combined exposures (protons + UVB and vice versa), neutron-gamma background (plate control) as well as observed in the PTB laboratory control. In total, four 4 cultures from 2 independent experiments have been analyzed per condition each represented by one column. UVB 400 J/m² followed by 0.5 Gy protons resulted in too less cells to reliably evaluate a dicentric frequency. *: no metaphase cells obtained, §: no dicentrics observed.

S9. Gene expression for HS27 and HaCaT cells exposed to UVB or protons as single stressors and stress genes expression in the co-exposed samples

<u>Fibroblasts</u>

Expression of genes of Hs27 fibroblasts exposed to UVB or protons is presented in Figure S9.1a and Figure S9.1b, while in Figure S9.1c several other stress genes that were found up-regulated at 48 h after exposure of Hs27 fibroblasts to protons, UVB, or to combined radiations are presented.

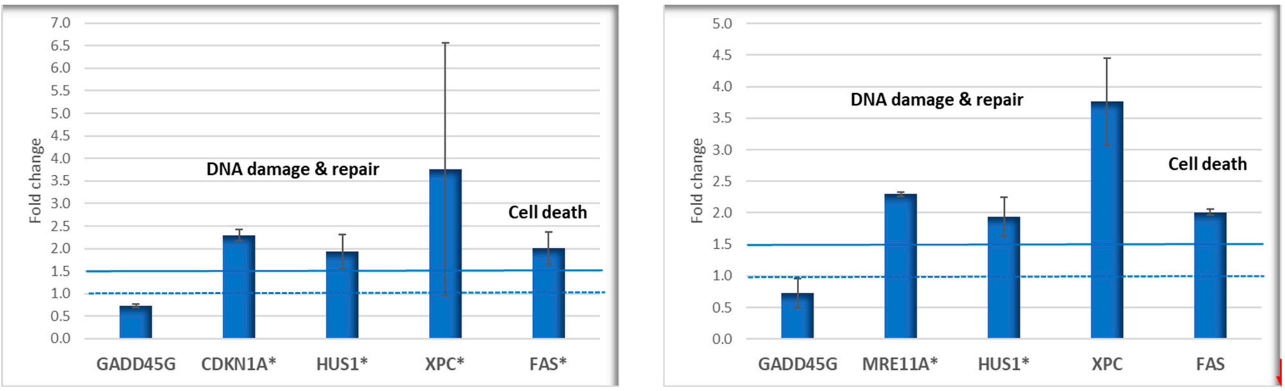

(a) (b)

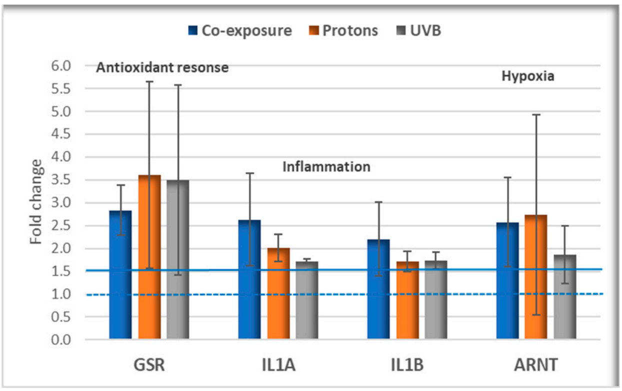

(c)

Figure S9.1. Stress genes with modified expression in human normal Hs27 fibroblasts exposed to 0.5 Gy protons or to 50 J/m² UVB applied as single stressors, or combined (proton irradiation followed by UVB exposure within approx. 20 min). Genes with mean fold change > 1.5 (gene up-regulation) or < 0.67 (gene down-regulation) were represented as mean value ± SD in 3 independent experiments. Genes with a significant fold change value in 2 out of 3 experiments and no significant effect in the third experiment were marked with *. The blue line is the low threshold for significant gene up-regulation, while the dashed blue line corresponds to "the no effect" case for evidencing gene down-regulation. (a) DNA damage & repair genes, and cell death genes in Hs27 fibroblasts exposed to protons; (b) DNA damage & repair genes, and death genes in Hs27 fibroblasts exposed to UVB; (c) other stress genes related to antioxidant response, inflammation, and hypoxia response in Hs27 fibroblasts exposed to protons or UVB, or co-exposed to combined radiations.

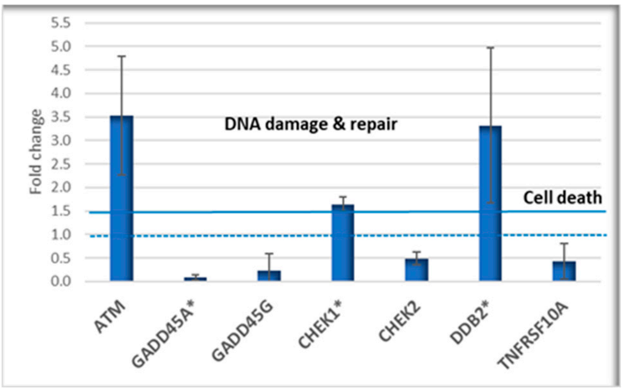

(a)

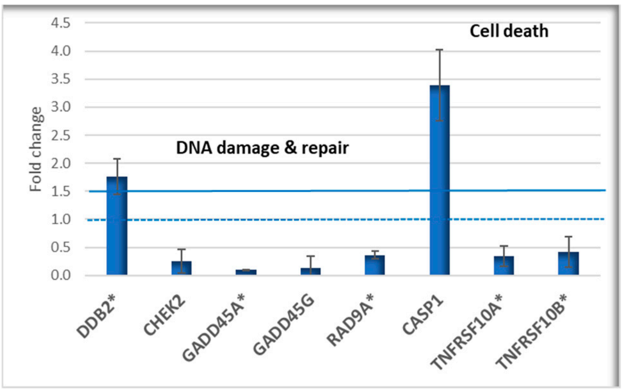

(b)

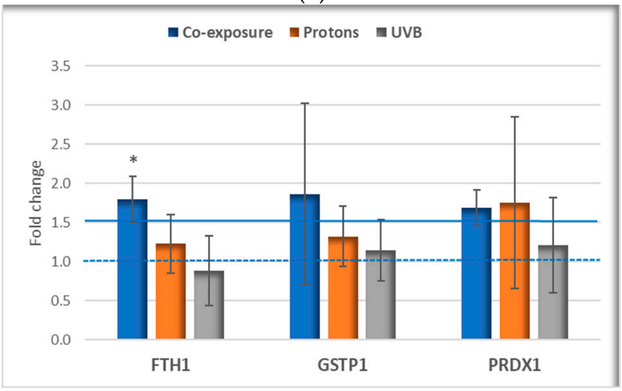

(c)

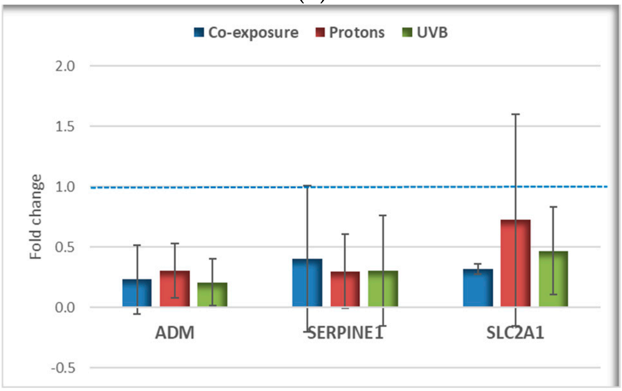

(d)

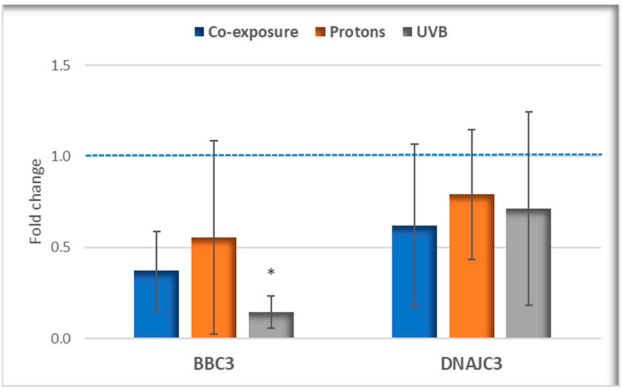

(e)

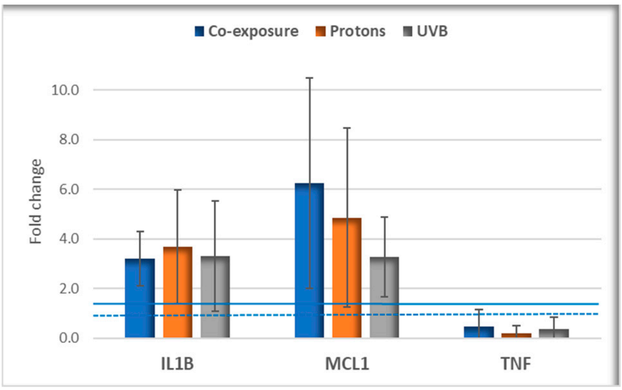

(f)

Figure S9.2. Stress genes with modified expression in human normal HaCaT keratinocytes exposed to 0.5 Gy protons or to 50 J/m² UVB applied as single stressors, or combined (proton irradiation followed by UVB exposure within approx. 20 min). Genes with mean fold change > 1.5 (gene up-regulation) or < 0.67 (gene down-regulation) were represented as mean value ± SD in 3 independent experiments. Genes with a significant fold change value in 2 out of 3 experiments and no significant effect in the third experiment were marked with *. The blue line is the low threshold for significant gene up-regulation, while the dashed blue line corresponds to "the no effect" case for evidencing gene down-regulation. (a) DNA damage and cell death genes in HaCaT keratinocytes exposed to protons; (b) DNA damage and cell death genes in HaCaT keratinocytes exposed to UVB; (c) Antioxidant response genes; (d) Hypoxia-related genes; (e) Unfolded protein response; (f) Inflammatory genes.

The expression pattern of stress genes in CRL 9855 exposed to 0.5 Gy protons or 50 J/m² UVB as single stressors, or co-exposed to protons and UVB is presented in Table S9.1.

Table S9.1 The expression pattern of stress genes in CRL 9855 exposed to 0.5 Gy protons or 50 J/m² UVB as single stressors, or co-exposed to protons and UVB. Expression changes were presented as fold change (FC) (mean value and SD for 2 independent experiments). FC values < 0.67 were considered significant for gene down-regulation.

	**Protons (0.5 Gy)**		**UVB (50 J/m²)**		**Protons+UVB**	
Stress pathway/genes	Mean FC	SD	Mean FC	SD	Mean FC	SD
**Cell death**						
CASP1	0.18	0.03	0.16	0.01	0.18	0.05
FAS	0.19	0.03	0.26	0.10	0.31	0.17
TNFRSF1A	0.84	0.20	0.85	0.08	0.49	0.00
ATG12	0.20	0.00	0.30	0.03	0.27	0.02
ATG5	0.18	0.04	0.42	0.19	0.25	0.10
ATG7	0.29	0.12	0.79	0.08	0.22	0.03
BECN1	0.30	0.08	0.44	0.03	0.32	0.13
BNIP3L	0.24	0.02	0.29	0.04	0.27	0.08
**DNA damage response**						
ATM	0.31	0.06	0.35	0.17	0.57	0.35
ATR	0.34	0.14	0.41	0.10	0.48	0.19

Unfolded protein response						
BID	0.24	0.04	0.36	0.04	0.39	0.10
CALR	0.26	0.10	0.31	0.03	0.19	0.11
HSP90B1	0.35	0.15	0.41	0.07	0.41	0.15
HSPA4	0.38	0.16	0.38	0.05	0.40	0.13
HSPA5	0.42	0.22	0.47	0.11	0.34	0.16
Oxidative stress						
FTH1	0.37	0.19	0.35	0.00	0.43	0.09
PRDX1	.57	0.08	0.63	0.04	0.74	0.04

The expression pattern of stress genes in CRL 9855 exposed to 0.5 Gy and 0.25 Gy protons is presented in Table S9.2.

Table S9.2. The expression changes of stress genes in CRL 9855 exposed to protons of 0.5 Gy or 0.25 Gy. Expression changes were presented as fold change (FC). FC values < 0.7 were considered significant for gene down-regulation. Results are expressed as mean value and SD. N=4 independent experiments for 0.5 Gy protons, and 2 independent experiments in the case of 0.25 Gy protons. In grey shadow are marked significant gene down-regulations in monocytes exposed to 0.25 Gy protons.

Pathway/genes	**Proton exposure**			
	**0.5 Gy**		**0.25 Gy**	
	Mean FC	SD	Mean FC	SD
**Cell death**				
GRB2	0.57	0.09	0.80	0.25
PVR	0.53	0.11	0.78	0.23
ATG7	0.45	0.21	0.39	0.06
BECN1	0.49	0.24	0.61	0.26
BNIP3L	0.49	0.31	0.66	0.33
**DNA damage**				
TP53	0.56	0.09	0.99	0.62
HUS1	0.49	0.28	0.80	0.09
MRE11A	0.59	0.28	1.00	0.01
Unfolded protein response				
ATF6	0.40	0.32	0.56	0.45
ATF6B	0.47	0.17	0.41	0.16
Antioxidant response				
GSR	0.55	0.13	0.69	0.07
Hypoxia response				

SLC2A1	0.55	0.10	0.82	0.37
VEGFA	0.64	0.32	1.04	0.06
Inflammation				
IL1A	0.45	0.31	0.78	0.07
Osmotic stress				
SLC5A3	0.51	0.10	0.95	0.57

UVB fluence-gene expression curves for stress genes in CRL 9855 monocytes are presented in Figure S9.3.

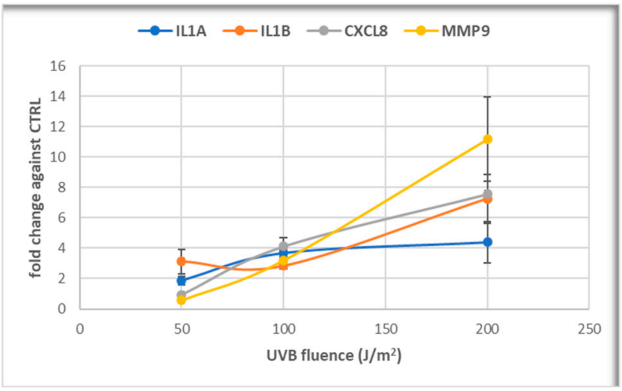

(**a**)

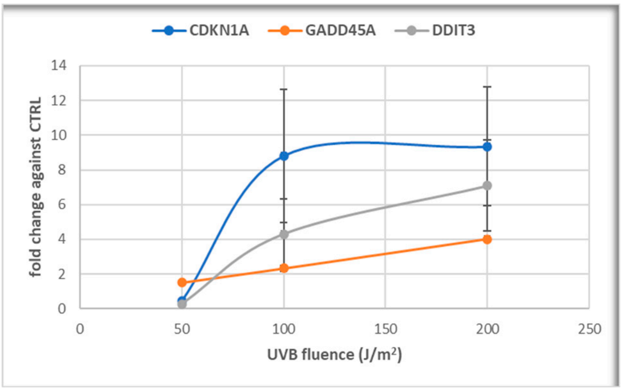

(**b**)

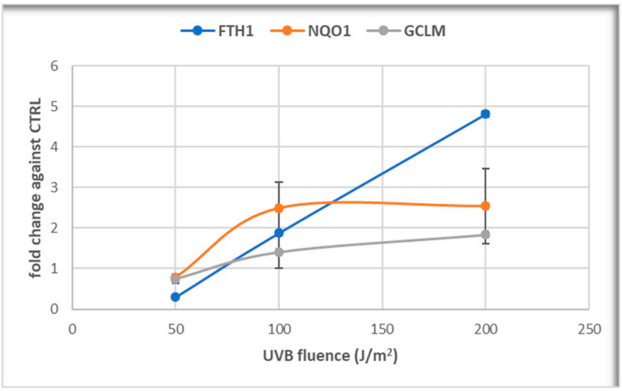

(c)

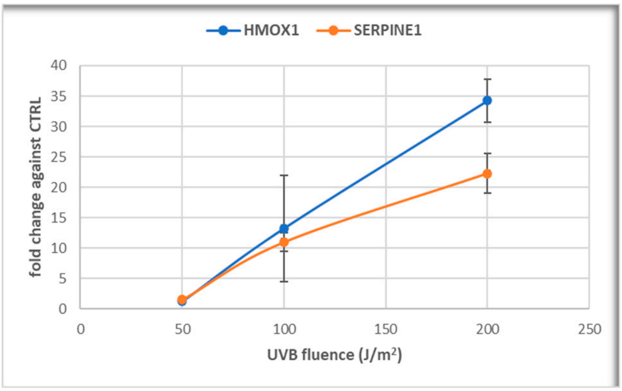

(d)

Figure S9.3. UVB fluence-gene expression curves for stress genes in CRL 9855 monocytes. Results are expressed as mean FC value ± SD for 2 independent experiments. a. Inflammation genes; b. DNA damage and endoplasmic reticulum stress genes; c, Antioxidant genes; d. Hypoxia genes.

S10. Survival of VH10 cells exposed to UVB and gamma rays

To investigate the survival of human fibroblasts, normal human skin fibroblast cells (VH10) were exposed to gamma radiation at a dose of 0.5 Gy (dose rate: 0.34 Gy/min) using a cesium-137 source (GammaCell-1000, Stockholm University). Additionally, UVB

irradiation was performed using a corona mini dose UV240T lamp (230 V, 50 Hz, 70 W) with a fluence of 1.4 W/m² for 66 seconds, corresponding to an energy dose of 100 J/m².

The VH10 cells were seeded in T25 flasks at a density of 20,000 cells/mL in Dulbecco's Modified Eagle Medium (DMEM, Sigma-Aldrich, Darmstadt, Germany) supplemented with 10% bovine serum (Sigma-Aldrich, Darmstadt, Germany) and 1% penicillin-streptomycin (Sigma-Aldrich, Darmstadt, Germany) 24 hours prior to irradiation. After treatment, the irradiated and control flasks were maintained in a cell culture incubator at 37°C with 5% carbon dioxide. All flasks were incubated for four days before trypsinization and manually counted total cell with the Counting chamber, Bürker-Türk (VWR, Lund, Sweden) to determine cell survival.

As shown in Figure S10, UVB irradiation at 100 J/m² resulted in a survival rate of 44.9% ± 4.9%, while gamma radiation at 0.5 Gy yielded a survival rate of 67.3% ± 16.8%. The combination treatment (UVB 100 J/m² + 0.5 Gy) further reduced survival to 38.3% ± 11.3%. The dose of 0.5 Gy and 100 J/m² were exposed base on the LD50 and cell survival.

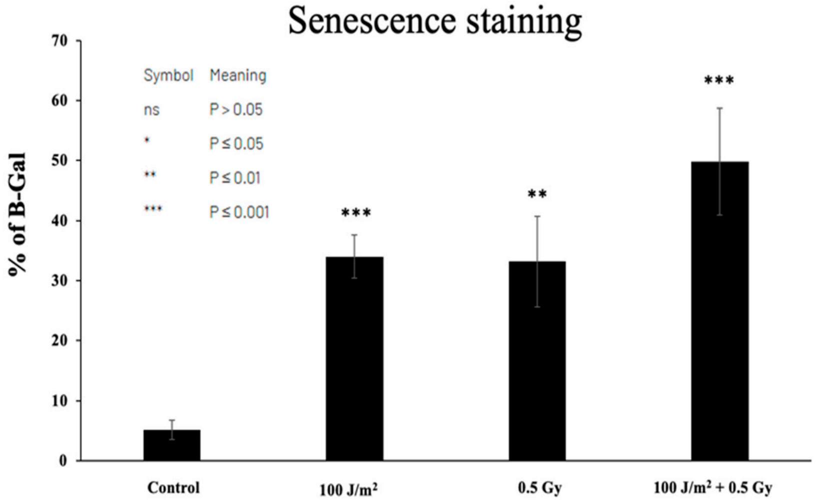

Figure S10. Relative Survival of VH10 cells following exposure to gamma radiation and/or UVB irradiation was assessed. Cell survival percentage was determined four days post-treatment, with the values normalized to the untreated control sample, which was set at 100%. Data are presented as the mean ± standard deviation from a minimum of three independent experiments. *: p<0.5 when treated samples was compared with nom-treated samples.

S11. Senescence of VH10 cells exposed to UVB and gamma rays

SA-β-gal staining was performed according to the protocol outlined by Dimri et al. [4]. One day before staining, cells were seeded into 6-well plates at a density of 20,000 cells per well. Each sample was prepared in duplicate, and the experiment was conducted in triplicate for individual experiment.

Cells were washed twice with PBS (Sigma-Aldrich, Darmstadt, Germany) and fixed at room temperature for 10 minutes using a solution of 3% formaldehyde and 2% sucrose (both from Sigma-Aldrich) in PBS. Following fixation, the cells were washed twice with PBS and incubated overnight at 37°C in SA-β-gal staining solution without carbon dioxide.

Before microscopic evaluation, the samples were washed again with PBS and briefly stained with 1% orcein in 45% acetic acid, followed by another PBS wash. Cells were maintained in 70% glycerol during scoring to preserve the staining and morphology for the identification of senescent cells. Approximately 500 cells per sample were manually examined, and the percentage of SA-β-gal-positive cells (characterized by blue-green

cytoplasmic staining) was calculated and orcein was visualized as red color for the normal cell under light microscope with magnification of 10x.

In the control group, 5.14% ± 1.61% of cells were senescent. UVB irradiation and gamma alone increased the percentage of senescent cells to 34.01% ± 3.58% and 33.16% ± 7.59%, respectively. The combined treatment of UVB and gamma radiation further elevated senescence to 49.82% ± 8.85%. Data represent the mean ± standard deviation of three independent experiments, with approximately 500 cells scored per sample. Error bars indicate the standard deviation.

The effect of UVB irradiation and mixed radiation (UVB + gamma radiation) on senescence in VH10 fibroblast cells was assessed using SA-β-gal staining, with the percentage of senescent cells calculated for each condition. Control cells (Figure S11-a) exhibited a low baseline level of senescence, with an average of 5.14% ± 1.61%. UVB irradiation (100 J/m²) (Figure S11-b) and gamma irradiation (Figure S11-c) significantly increased the percentage of senescent cells ($p<0.001$ compared to control). The mixed radiation treatment (UVB + 0.5 Gy gamma radiation) (Figure S11-d) further increased the percentage of senescent cells to 49.82% ± 8.85%, representing a highly significant difference compared to both the control ($p<0.001$). These results demonstrate that both UVB, gamma radiation and mixed radiation treatments induce significant cellular senescence in VH10 fibroblast cells, with the combination of UVB and gamma radiation causing the highest level of senescence (Figure S11-e). This suggests a synergistic effect of the combined treatments in promoting cellular senescence.

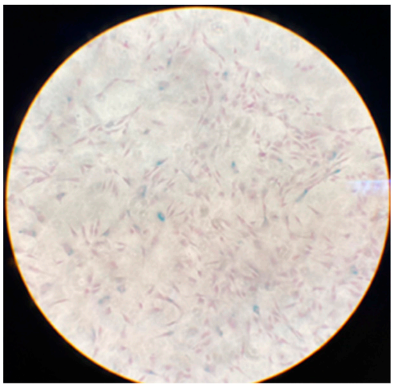

(**a**)

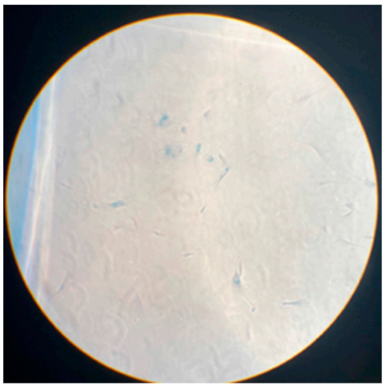

(**b**)

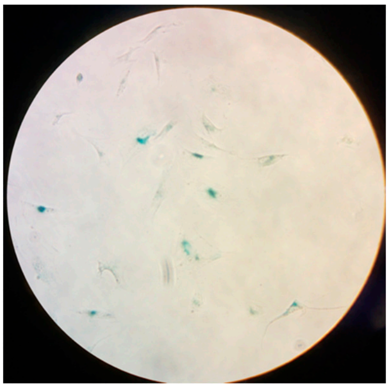

(c)

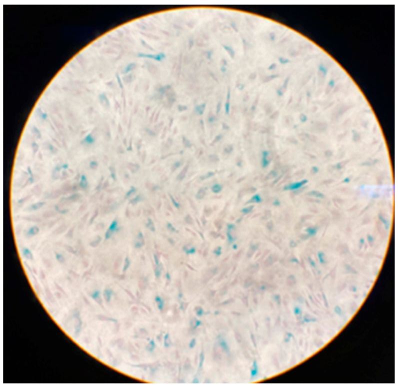

(d)

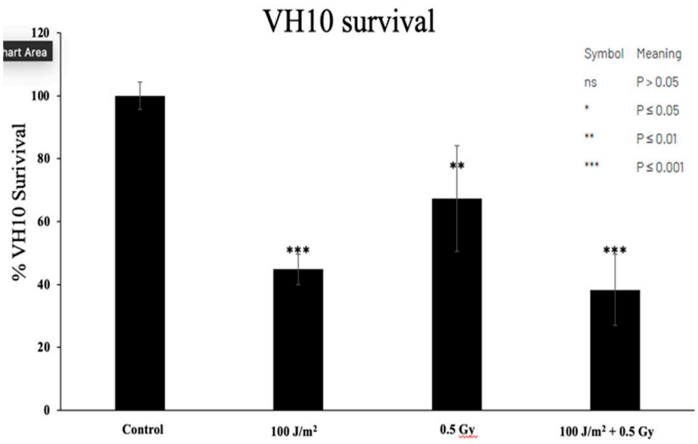

(e)

Figure S11. Senescence-associated β-galactosidase (SA-β-gal) staining (characterized by blue-green cytoplasmic staining) and orcein (red to visualize non-senescence cells) of VH10 fibroblast cells was conducted under various conditions to assess senescence levels. (a) Untreated cells (Control, A) displayed baseline SA-β-gal staining; (b) Cells exposed to UVB irradiation (100 J/m^2; (c) or gamma radiation (0.5 Gy, C) exhibited increased SA-β-gal staining, and the mixed radiation (100 J/m^2 + Gamma radiation; (d) Images were captured using light microscopy to visualize staining patterns at 10x magnification; (e) The percentage of senescent VH10 fibroblast cells was determined following exposure to UVB (100 J/m^2), gamma radiation (0.5 Gy), and a combination of both, using SA-β-gal staining as a marker of senescence. Data are presented as the mean ± standard deviation from a minimum of three independent experiments.

S12. Immunofluorescence protocol variations for different cell lines

For monocytes following irradiation with protons and/or UVB, cells were immediately returned to a 37°C incubator, and were harvested thereafter at various time points for the γH2AX assay. H2AX phosphorylation detection was performed by using a modification of the protocol in the main text. Briefly, cells were centrifuged on microscope slides using a Cytospin ROTANTA 460/460R centrifuge (Hettich, Tuttlingen, Germany). All cells were fixed for 15 min at room temperature (RT) with 3% paraformaldehyde (F8775, Sigma-Aldrich, Darmstadt, Germany) and 2% sucrose (A2211, Applichem GmbH, Darmstadt, Germany) in phosphate buffered saline (PBS, 18912-014, Gibco, Grand Island, NY, USA), and then were washed thrice with PBS. Cells were thereafter permeabilized for 10 min with 0.5% Triton X-100 (X100, Merck, Saint Louis, MO, USA) in 100 mM Tris-HCL pH 7.4 (A4263, Applichem GmbH, Darmstadt, Germany) and 50 mM EDTA pH 8 (A4982, AppliChem GmbH, Darmstadt, Germany) in distilled water, washed again three times with PBS, and blocked overnight at 7°C with 0.5% bovine serum albumin (BSA, A7906, Sigma-Aldrich, Darmstadt, Germany) and 0.2% gelatin from cold water fish (G7041, Sigma-Aldrich, Darmstadt, Germany) in PBS. Cells were incubated with an antibody against histone H2AX (p S139) (NB100-384, Novus Biologicals, Abingdon, UK) in 0.5% BSA and 0.2% gelatin in PBS for 90 min at RT, and then were washed three times with PBS. A 90 min incubation at RT with goat anti-rabbit IgG H&L cross-absorbed secondary antibody labeled with Rhodamine Red-X (R-6394, Thermo Fisher Scientific, Waltham,

MA, USA) in 0.5% BSA and 0.2% gelatin in PBS followed. After three times washing with PBS, DNA was counterstained with ProLong Gold Antifade Reagent with 4′6-diamidino-2-phenylindole (DAPI) (8961, Cell Signaling Technology Inc, Danvers, MA, USA), and cells were shielded with 22x22 mm coverslips, avoiding air bubbles trapping.

At least two independent experiments were conducted for each experimental condition in all cell lines. Microscope slides were analyzed using a Zeiss Axioskop-2 fluorescence microscope with ISIS software. More than 100 cells/slides were analyzed with the JCountPro and JQuantPlus image processing software.

For the detection of γH2AX foci in VH10 cells a slightly modified protocol was followed. Cells were fixed with 3% paraformaldehyde containing 2% sucrose (both from Sigma-Aldrich, Darmstadt, Germany) for 10 minutes at room temperature. Permeabilization was performed using 0.2% Triton X-100 (Sigma-Aldrich) for 10 minutes at room temperature. After permeabilization, the cells were washed three times with PBS and incubated at 37°C for 1 hour with the primary antibody anti-phospho-H2AX (Millipore, Billerica, MA, USA) diluted 1:800 in PBS supplemented with 2% Bovine Serum Albumin Fraction V (BSA-V, Sigma-Aldrich). Following primary antibody incubation, the cells were quickly washed with PBS and incubated for 30 minutes at 37°C with a secondary antibody, goat anti-mouse IgG conjugated to FITC (Sigma-Aldrich), diluted in PBS containing 2% BSA-V. After washing with PBS, the cells were stained with 4′,6-diamidino-2-phenylindole (DAPI, Sigma-Aldrich) in PBS for 10 minutes. The coverslips were carefully removed from the Petri dish, mounted on microscope slides using Vectashield® Antifade Mounting Medium (Vector Laboratories, Peterborough, UK), and sealed. Slides were analyzed using a Nikon Eclipse E800 fluorescence microscope at 100× magnification. Images of at least 50 cells were captured using a CCD camera (CoolCube1, Metasystems, Altlussheim, Germany) and processed imaged to TIFF file format. The number of γH2AX foci per nucleus was analyzed with the JCountPro and JQuantPlus image processing software.

References

1. Groom, D.E.; Mokhov, N.V.; Striganov, S.I. MUON STOPPING POWER AND RANGE TABLES 10 MeV–100 TeV. *Atomic Data and Nuclear Data Tables* **2001**, *78*, 183-356, doi:https://doi.org/10.1006/adnd.2001.0861.
2. Sato, T. Analytical Model for Estimating Terrestrial Cosmic Ray Fluxes Nearly Anytime and Anywhere in the World: Extension of PARMA/EXPACS. *PloS one* **2015**, *10*, e0144679, doi:10.1371/journal.pone.0144679.
3. Hernández, A.; Endesfelder, D.; Einbeck, J. Biodose Tools: an R shiny application for biological dosimetry. **2023**, *99*, 1378-1390, doi:10.1080/09553002.2023.2176564.
4. Dimri, G.P.; Lee, X.; Basile, G.; Acosta, M.; Scott, G.; Roskelley, C.; Medrano, E.E.; Linskens, M.; Rubelj, I.; Pereira-Smith, O., et al. A biomarker that identifies senescent human cells in culture and in aging skin in vivo. *Proceedings of the National Academy of Sciences of the United States of America* **1995**, *92*, 9363-9367, doi:10.1073/pnas.92.20.9363.
